# Effects of spaceflight aboard the International Space Station on mouse estrous cycle and ovarian gene expression

**DOI:** 10.1038/s41526-021-00139-7

**Published:** 2021-03-12

**Authors:** Xiaoman Hong, Anamika Ratri, Sungshin Y. Choi, Joseph S. Tash, April E. Ronca, Joshua S. Alwood, Lane K. Christenson

**Affiliations:** 1 Department of Molecular and Integrative Physiology, University of Kansas Medical Center, Kansas City, KS USA; 2 KBR, NASA-Ames Research Center, Moffett Field, CA USA; 3 Space Biosciences Division, NASA-Ames Research Center, Moffett Field, CA USA; 4 Department of Obstetrics & Gynecology, Wake Forest Medical School, Winston-Salem, NC USA

**Keywords:** Physiology, Developmental biology

## Abstract

Ovarian steroids dramatically impact normal homeostatic and metabolic processes of most tissues within the body, including muscle, bone, neural, immune, cardiovascular, and reproductive systems. Determining the effects of spaceflight on the ovary and estrous cycle is, therefore, critical to our understanding of all spaceflight experiments using female mice. Adult female mice (*n* = 10) were exposed to and sacrificed on-orbit after 37 days of spaceflight in microgravity. Contemporary control (preflight baseline, vivarium, and habitat; *n* = 10/group) groups were maintained at the Kennedy Space Center, prior to sacrifice and similar tissue collection at the NASA Ames Research Center. Ovarian tissues were collected and processed for RNA and steroid analyses at initial carcass thaw. Vaginal wall tissue collected from twice frozen/thawed carcasses was fixed for estrous cycle stage determinations. The proportion of animals in each phase of the estrous cycle (i.e., proestrus, estrus, metestrus, and diestrus) did not appreciably differ between baseline, vivarium, and flight mice, while habitat control mice exhibited greater numbers in diestrus. Ovarian tissue steroid concentrations indicated no differences in estradiol across groups, while progesterone levels were lower (*p* < 0.05) in habitat and flight compared to baseline females. Genes involved in ovarian steroidogenic function were not differentially expressed across groups. As ovarian estrogen can dramatically impact multiple non-reproductive tissues, these data support vaginal wall estrous cycle classification of all female mice flown in space. Additionally, since females exposed to long-term spaceflight were observed at different estrous cycle stages, this indicates females are likely undergoing ovarian cyclicity and may yet be fertile.

## Introduction

Spaceflight microgravity is known to impact numerous physiological systems. These include cardiovascular changes, decreased bone mineral density, loss of muscle tone, adipogenesis, insulin resistance, vision and vestibular disturbances, altered fluid and electrolyte balance, and altered kidney function to name a few^1^. Many, if not all, normal homeostatic processes occurring within mammals, both male and female, are dramatically impacted by gonadal hormones. Indeed, the reproductive system is often considered one of the best markers of overall health and fitness of an animal^2^. However, research is limited regarding the effects of spaceflight on the reproductive system and its consequences on normal physiology^3,4^.

The ovarian steroid hormones, estrogen and progesterone, play a significant role in regulating a myriad of physiological functions, and are subject to cyclic changes based on the hypothalamic/pituitary regulation of ovarian folliculogenesis^5^. Moreover, ovarian steroidogenic output can be dramatically impacted by stress as well as other internal homeostatic processes^6^. In cycling rodents, the antral follicle is the primary steroidogenic tissue within the ovary, unless mating occurs at which time the corpus luteum becomes the major producer of progesterone. The mural granulosa cells and the vascularized theca cells within the antral ovarian follicles are responsible for the enzymatic conversion of cholesterol to its ultimate endpoint product estradiol. In addition to steroidogenesis, these follicular cells also carry out the critical missions of nurturing the enclosed growing oocyte, as well as facilitating the process of ovulation, or expulsion of oocyte, and subsequent formation of the ephemeral corpus luteum. Cyclic and temporal changes in expression of steroidogenic pathway proteins and other critically important ovarian genes known to play critical roles in ovulation, oocyte development and corpus luteum development and function are well known and may be evaluated to provide information regarding ovarian function. Understanding these molecular changes is vital for informing female reproductive health during and after spaceflight.

To date, studies of ovarian function and fertility in mice exposed to microgravity during NASA Space Shuttle flights were of short duration (<12 days) and, importantly, all included the effects of live reentry prior to collection of tissues and analysis of animal behavior^3,7^. Similarly, pregnant female rats flown on Cosmos 1514 (1982), NASA-NIH Rodent (R)1 (STS-66 in 1994), and NASA-NIH R2 (STS-70 in 1995) were of short duration (4.5–11 days) and had live animal return^8–10^. Notably, in the pregnant rats no effects of spaceflight on healthy and atretic ovarian antral follicle populations, fetal wastage in utero, plasma concentrations of progesterone and luteinizing hormone (LH) or pituitary content of follicle stimulating hormone (FSH) were noted^8^. Spaceflight, however, significantly increased plasma concentrations of FSH and decreased pituitary content of LH analyzed postpartum (day 22–23;^8^). In these prior studies, it is not possible to separate the effects of microgravity from that of reentry with the associated hypergravity, turbulence, and the likely ensuing stress responses linked to these events. Moreover, these studies while evaluating maintenance of pregnancy, provide no information regarding the fertility of these animals and their ability to exhibit normal estrous cycles and ovarian function. The current investigation provides insights into the endocrine status and ovarian cyclicity of female mice following an extended period of exposure to microgravity in the absence of reentry effects. Addressing the effects of extended duration spaceflight on endocrine status are significant and of high impact to long-term female astronaut health before and after flight, interpretation of spaceflight findings derived from female mice, and for long-term plans for multigenerational mammalian studies in spaceflight.

## Results

### Estrous cycle staging

**Fig. 1:**
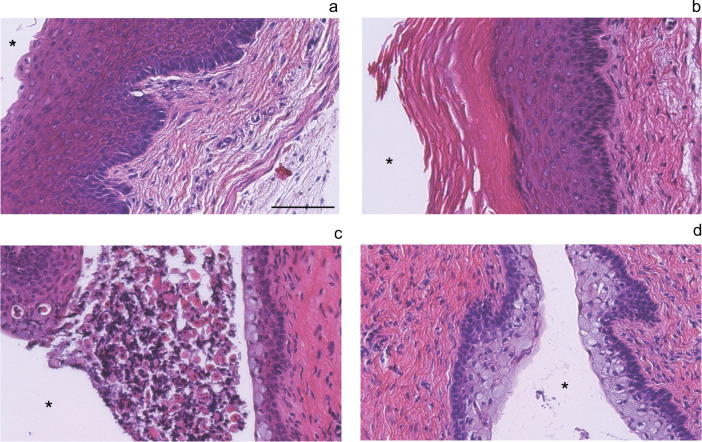
Hematoxylin and eosin staining of vaginal walls used for estrous cycle staging. Vaginal wall estrous cycle staging was completed on all mice (*n* = 40). Representative sections from two flight and two habitat control mice depict each stage of the estrous cycle **a** proestrus, **b** estrus, **c** metestrus, and **d** diestrus. *Marks the vaginal lumen. Images were taken at ×20 using brightfield microscopy; scale bar measures 100 μm.

Estrous cycle stage for each mouse was determined by three independent investigators blinded to treatment group categories. Figure 1 shows representative H&E stained cross sections of vaginal wall tissue from the individual flight and habitat control animals following collection after the second carcass thaw during March of 2016. Each stage of the estrous cycle is depicted in Fig. 1. Proestrus is characterized by the presence of a thick layer of stratified squamous epithelium, with an absence of luminal cells and any cornified epithelial cells (Fig. 1a). Estrous stage animals exhibit a stratified squamous epithelium with a layer of cornified epithelial [red eosin stained cells above the nucleated stratified squamous epithelial cells (Fig. 1b)]. Often, this cornified layer is delaminated from the underlying epithelial cells. In metestrus, leukocytes appear and traverse the nucleated epithelial layer to reach the delaminated mass of cornified stratified squamous epithelium in the lumen (Fig. 1c). During diestrus, the vaginal wall is characterized by columnar mucified cells next to a thin layer of nucleated epithelium (Fig. 1d). Staging of the mice indicated that the majority of the animals in the baseline control group (6/9), the vivarium control group (5/8), and the flight group (6/10) were in estrus (Table 1). In comparison, habitat control mice presented at all stages of the cycle in approximately equal amounts (*n* = 2 or 3/stage). Flight group mice presented at estrus (*n* = 6) and metestrus (*n* = 4; Table 1).

**Table 1:** Number of mice at each stage of the estrous cycle (*n* = 10/group).

	Proestrus	Estrus	Metestrus	Diestrus	ND
Baseline	2	6	0	1	1
Vivarium	3	5	0	0	2
Habitat	2	3	2	3	0
Flight	0	6	4	0	0

*ND* not determinable.

### Ovarian RNA quality and tissue steroid levels

**Fig. 2:**
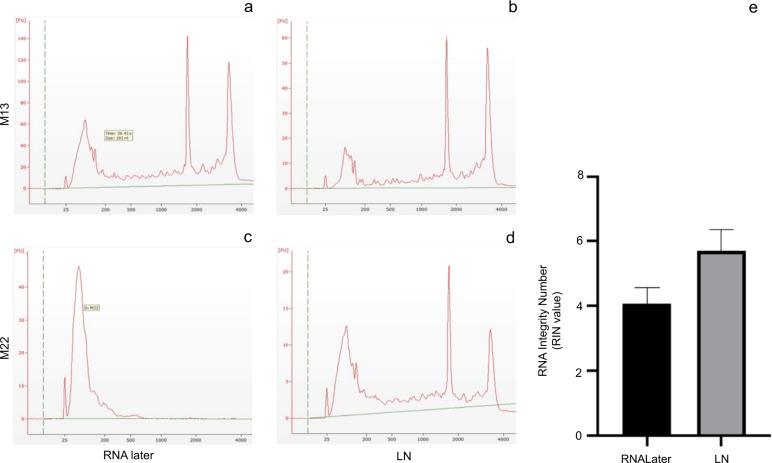
RNA quality of liquid nitrogen snap-frozen and RNAlater preserved ovarian tissues. Representative electropherograms from 2 animals (M13 vivarium control **a**, **b** and M22 flight **c**, **d**) perserved in RNAlater (**a**, **c**) or LN (**b**, **d**). **e** Histogram of RIN values from analysis of paired LN and RNALater samples (*n* = 12; 4BL, 4VC, 2HC, 2FL). *Means ± SEM RIN values were significantly (*p* ≤ 0.05) different based on the preservation method.

**Fig. 3:**
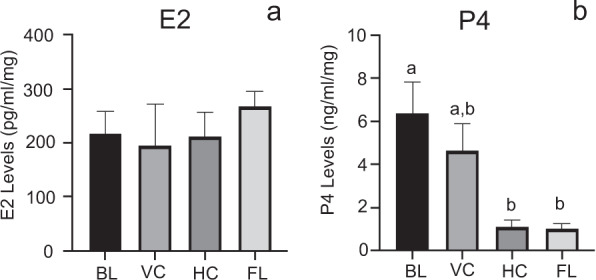
Whole ovarian tissue estrogen and progesterone concentrations. **a** Estradiol (E2; pg/ml/mg tissue) and **b** progesterone (P4; ng/ml/mg tissue) levels for all mice within a treatment group (*n* = 8-9/treatment; 9BL, 8VC, 9HC, 9FL). ^a,b^Means ± SEM with different superscripts are significantly different (*p* ≤ 0.05).

Ovarian RNA quality varied appreciably depending upon whether it was flash-frozen in LN or placed in RNAlater (Fig. 2). Representative electropherograms from the Agilent 2100 are shown for 2 mice collected in either RNAlater or LN. Overall, RNA quality as determined by Agilent RNA integrity numbers (RIN values) from tissues frozen in LN were greater (*p* < 0.05) in every animal, and overall averaged around 5.71 ± 0.12 compared to 4.08 ± 0.11 for RNAlater preserved ovarian tissue (Fig. 2b). To evaluate ovarian hormone production without access to serum, whole ovarian tissue steroid levels were determined. Ovarian tissue E2 levels were not significantly different across experimental groups (Fig. 3a). In contrast, whole ovarian tissue P4 levels were significantly lower in the habitat control (*p* = 0.005) and flight mice (*p* = 0.013) in comparison to baseline control mice (Fig. 3b). Due to insufficient animal numbers at individual stages within treatment groups, we were unable to evaluate stage effects alone and stage-treatment interactions.

### Ovarian tissue gene expression analyses

**Fig. 4:**
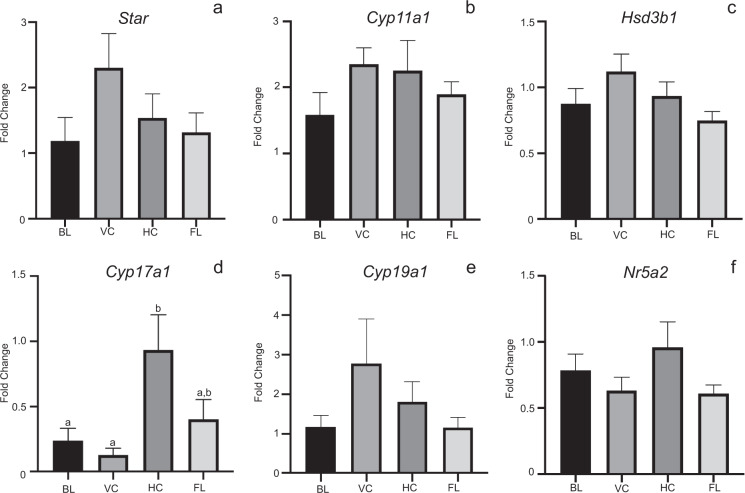
Whole ovarian tissue steroidogenic gene expression was not altered by spaceflight. Histograms depict relative fold change (ΔΔCt method) for **a** *Star*, **b** *Cyp11a1*, **c** *Hsd3b1*, **d** *Cyp17a1*, **e** *Cyp19a1*, and **f** *Nr5a2* following normalization to 18S; *n* = 8–10/treatment group; 10BL, 10VC, 8HC, 10FL). ^a,b^Means ± SEM with different superscripts are significantly different (*p* ≤ 0.05).

Quantitative RT-PCR analysis of critical proteins involved in ovarian function and steroidogenesis were compared across treatment groups irrespective of stage of the cycle (Fig. 4, Supplementary Fig. 2). Spaceflight had no demonstrable effect on gene expression of any of the key enzymatic steps of steroidogenesis or mitochondrial cholesterol uptake expression. Ovarian *Cyp17a1* mRNA levels were elevated (*p* ≤ 0.05) in mice housed in the habitat maintained on the ground (HC) when compared to baseline and vivarium controls but were not different from flight animals that were housed in the habitat on the ISS (Fig. 4). Analysis of other critically important ovarian genes involved in steroidogenic production and action, including estrogen receptors (*Esr1* and *Esr2*), scavenger receptor class B type 1 (*Scarb1*), luteinizing hormone receptor (*Lhcgr*) as well as oocyte-specific proteins, growth differentiation factor 9 (*Gdf9*), zona pellucida glycoprotein 3 (*Zp3*) showed no differences across the treatment groups (Supplementary Fig. 1).

**Fig. 5:**
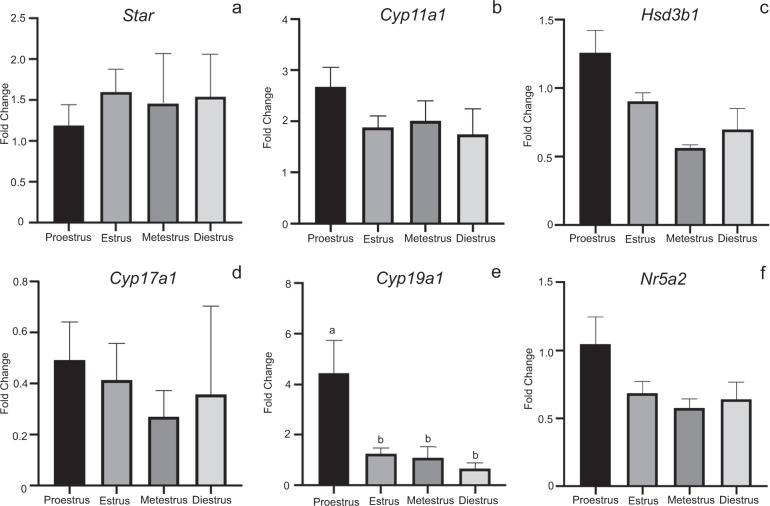
Whole ovarian tissue steroidogenic gene expression by stage of estrous cycle. Histograms depict relative fold change (ΔΔCt method) for **a** *Star*, **b** *Cyp11a1*, **c** *Hsd3b1*, **d** *Cyp17a1*, **e** *Cyp19a1*, and **f** *Nr5a2* following normalization to 18S; *n* = 8–10/treatment group; 10BL, 10VC, 8HC, 10FL). ^a,b^Means ± SEM with different superscripts are significantly different (*p* ≤ 0.05).

**Fig. 6:**
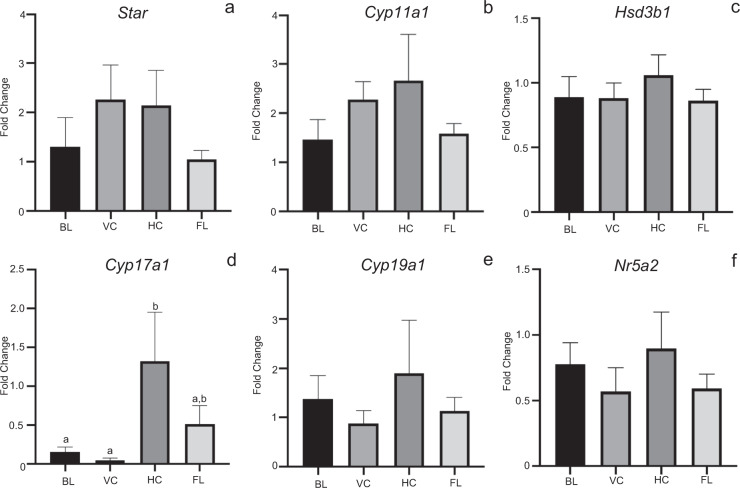
Whole ovarian tissue steroidogenic gene expression at estrus across all four treatment groups. Histograms depict relative fold change (ΔΔCt method) for **a** *Star*, **b** *Cyp11a1*, **c** *Hsd3b1*, **d** *Cyp17a1*, **e** *Cyp19a1*, and **f** *Nr5a2* following normalization to 18S; *n* = 3–6/treatment group; 6BL, 5VC, 3HC, 6FL). ^a,b^Means ± SEM with different superscripts are significantly different (*p* ≤ 0.05).

Comparison of ovarian gene expression at different stages of the estrous cycle did not vary significantly with exception of proestrus, where P450 aromatase (*Cyp19a1*) expression was elevated (*p* ≤ 0.05) ~4-fold compared to all other stages (Fig. 5). Lastly, as each group of animals had at least three animals staged at estrus, we compared the gene expression across the four treatment groups at this stage (Fig. 6). Levels of steroidogenic acute regulatory protein (*Star*), P450 cholesterol side chain cleavage (*Cyp11a1*), 3β-hydroxysteroid dehydrogenase (*Hsd3b1*), *Cyp19a1*, and liver receptor homolog-1 (*Nr5a2*) expression did not change appreciably across experimental conditions. Expression of *Cyp17a1* under baseline and vivarium control conditions were significantly decreased (*p* < 0.05) compared to habitat control conditions (*p* < 0.05). Ovarian *Cyp17a1* levels in flight animals in estrus were not notably different from estrus stage animals in the other experimental conditions (Fig. 6). Examination of other critical ovarian genes during estrus showed no significant changes in expression levels across control and flight conditions (Supplementary Fig. 2).

## Discussion

Rodent Research-1 (RR-1), a Validation mission launched aboard SpaceX’s fourth cargo resupply mission, was the first to transport rodents to the International Space Station (ISS) in an unmanned commercial vehicle. Lasting 37 days in microgravity, RR-1 was, at the time, the longest space rodent study conducted by NASA. In-flight studies of RR1 mouse behavior and post-flight physiology revealed the Validation mice showed no overt physiological signs of chronic stress or compromised health or welfare^11^. Moreover, mice remained highly active and mobile throughout the experiment, engaging in feeding, drinking, self-grooming, huddling, and social interactions^12^. Our results provide detailed consequences of long-term spaceflight microgravity on rodent estrous cycle, ovarian steroidogenesis, and gene expression. Notably, previous observations examining the potential impacts of microgravity on ovarian function and its target tissue the uterus were of a short-term nature and had the confounding effects of reentry prior to tissue collection^3,9,13^. It is well known that estrous cycle staging in female rodents can be influenced by a variety of factors such as age, light, temperature, humidity, ambient noise, nutrition, and social relationships^14^. Indeed, mice that are group housed exhibit greater estrous cycle irregularities, than those individually housed^15,16^. Nonetheless, many labs using contemporary strains of mice are able to stage mice in group housing conditions, albeit it may not be as ideal as single housed mice^17–19^. Again, group housing was necessitated by animal husbandry concerns and limitations of the NASA’s flight hardware. Our results point to an ability of female mice to exhibit estrous cycles after an extended duration in space, as evidenced by our observation that flight animals exhibited two different stages of the cycle (estrus and metestrus). Typically, under conditions of exogenous treatments/stress, mice may exhibit constant vaginal estrus or an extended or constant diestrus^14,20^, but not metestrus. In contrast to mice, rats exhibit a greater synchrony when group housed, making it difficult to extrapolate finding across these two species^21^.

While measuring true estrous cyclicity in space is logistically daunting due to the intensive daily monitoring required, we posit that the analysis of the vaginal wall provides a robust methodology that can indicate the estrous stage, and thus the endocrine status of the animal at the moment of sacrifice. Indeed the morphology of these twice frozen and thawed vaginal wall tissues rivaled that of freshly isolated and fixed tissues from contemporary animals collected in our laboratory (data not shown) and in those laboratories who pioneered the use of vaginal wall histology as a mechanism to track cyclicity^20,22,23^.

Ovarian tissue steroid levels are also not commonly measured, as most investigators use serum levels of estrogen and progesterone as a measure of ovarian steroidogenic activity. Again, in animals exposed to microgravity, the ability to collect serum at the time of sacrifice or longitudinally over time is severely restricted logistically. We, therefore, analyzed whole ovarian tissue levels of estrogen and progesterone. We detected no striking difference in tissue estrogen concentrations across the four treatment groups, and it should be noted that serum estrogen levels in rodents also do not exhibit wide variations across the cycle, often ranging from ND to 8–50 pg/ml in total^24–27^. In contrast, we did observe that flight and habitat control animals had lower overall P4 levels in ovarian tissue. One might predict this as these two groups had greater numbers of mice in metestrus and diestrus, in total 4 and 5 for flight and habitat groups, respectively. During both of these stages of estrous cycle the ovary has reduced steroidogenic activity^26–28^. This was in contrast to the vivarium and baseline control groups, which both had 8 animals each in proestrus and estrus in total, stages associated with increased ovarian follicular steroidogenesis. We recognize that it would have been ideal to examine the interaction of estrous cycle stage on ovarian steroidogenesis as well as ovarian gene expression, but due to the insufficient numbers of animals at individual stages, we were unable to do so. It is worth noting the experimental design for RR1 as a hardware demonstration flight determined how tissues were preserved as well as group sizes and thus were beyond our control. Whole ovarian gene expression analyses were also dictated by the RR1 design. This type of analysis is highly confounded as whole ovarian tissue contains an abundance of stromal tissue and smaller follicles, which predominate over the few selected follicles that become highly steroidogenic. Thus, when whole ovarian RNA is used, it was not surprising to see no major differences in ovarian gene expression across the treatment groups or even during different stages of the cycle. Future studies need to take into consideration this limitation, and collect and fix tissues for analysis, so they can be properly analyzed using in situ hybridization/RNAscope and immunohistochemistry. *Cyp17a1*, the enzyme located in the theca cell compartment of the ovarian follicle and responsible for androgen production was elevated in the habitat control animals. This difference was however, manifested primarily in two of the three HC animals in estrus.

Safeguarding RNA tissue quality is acutely dependent on tissue storage methods, which ideally would minimize RNase activity throughout the process. Tissue collection and preservation during spaceflight presents a unique challenge in the analysis of these valuable specimens. Our current analyses of the right and left ovaries processed in RNALater or snap-frozen in liquid nitrogen (LN) indicated that LN preserved tissues always had greater RIN values when compared to those of RNALater. Previous studies utilizing RR1 RNAlater preserved spleen tissues showed higher RIN values than those observed here^29^. This may be due to a time of collection aspect with respect to spleen vs ovary following thawing of the carcass, or just to tissue differences in RNA susceptibility to the freeze/thaw. We note that freshly isolated ovarian tissues that were snap-frozen in LN and then simultaneously processed with the RR1 samples yielded RIN values >9.

In conclusion, our analyses of ovarian tissues and vaginal wall tissues of mice exposed to extended periods of microgravity and sacrificed in space indicate that these female mice were exhibiting estrous cyclic activity. These studies point positively to the ability of these animals to either continue or regain estrous cycle activity during extended microgravity exposure. We posit that vaginal wall estrous cycle activity be collected and provided to investigators to include as covariate on all female mice used in future space missions, much the same as weight and age. The current studies also provide critical logistical information regarding tissue collection, particularly for organs such as the ovary and uterus, which are composed of highly dynamic tissues (follicles, corpora lutea, endometrium), we recommend that they be preserved primarily in fixatives or in a manner where tissue/cell specific aspects can be determined afterward. Lastly, the ability of these females to exhibit estrous cycle activity provides a tractable mammalian model system to test whether fertility is actually impacted by microgravity. Reproductive cycles characterized by alterations in hormone levels can exert significant effects on experimental outcomes, including emotion and the response to environmental stressors^30^. Determining the effects of spaceflight on estrous cyclicity is thus critical to making accurate interpretations of experimental endpoints for all spaceflight experiments using female mice.

## Methods

### Animals

Forty 12-week old C57BI/6J female mice (Jackson Lab, Bar Harbor, ME) were selected based on similar body weights for 4 experimental groups (16-week old at launch, *n* = 10/group): baseline control, vivarium control, habitat control, and flight. Flight mice were launched on SpaceX-4 (Rodent Research 1) on September 21, 2014, as part of NASA’s Validation study. Details of RR1 mission timeline (i.e., mouse pre-adaptation to cages, food) as well as mouse spaceflight hardware details are available^11,12,31^. Briefly, vivarium control mice were maintained in standard mouse cages (5 mice/cage) for the duration of spaceflight at Kennedy Space Center. Habitat control mice were housed in a Rodent Research Transporter and Habitat Hardware on Earth for durations consistent with flight times at the same density of mice as those in flight (*n* = 10/habitat). The habitat control was placed in the ISS Environmental Simulator at KSC on a 4-day delay to mimic flight temperature, CO_2_, and humidity conditions on the ISS for the duration of spaceflight. Baseline control mice were euthanized one day after launch and then partially dissected prior to freezing of the animal carcasses mimicking the procedures to be conducted on flight and for all other animals at the conclusion of experiment. Flight mice were exposed to microgravity for a total of 37 days (33 days on ISS and 4 days in the Dragon Capsule). Mice were euthanized by injection of Euthasol followed by cervical dislocation and immediately fast frozen intact (*n* = 8) or partially dissected (*n* = 2) prior to carcasses being frozen. Mice carcasses were frozen using pre-chilled Ice Bricks prior to transfer to the Minus Eighty Degree Laboratory Freezer (MELFI) aboard the ISS. All ground mice were processed similarly. Flight mice carcasses returned to Earth February 2015 aboard the SpaceX CRS-5 and were then maintained along with the control mice frozen at the Biospecimen Sharing Program (BSP) at the Ames Research Center until dissection^11^. All animal procedures performed were approved by the Institutional Animal Care and Use Committees (IACUC) for flight at the NASA Ames Research Center (ARC) and the Kennedy Space Center (KSC) and the methods were carried out in accordance with relevant guidelines and regulations^11^.

### Tissue collection

Female reproductive tissues (ovary and uterus) were collected from all carcasses at about 35–45 min after removal from the −80 °C freezer. One ovary was placed into RNAlater (Thermo Fisher Scientific, Waltham, MA) and the other was flash-frozen in liquid nitrogen (LN). Similarly, the right and left uterine horns were placed in RNAlater or snap-frozen in LN; uterine tissues are part of an independent study by another investigator and will not be further described here. Tissues preserved in RNAlater were kept at 4 °C for 2 days then frozen and stored at −80 °C, tissues snap-frozen in liquid nitrogen were also stored at −80 °C. Following primary tissue dissections in April of 2015, the remaining carcass was refrozen and stored at −80 °C. In March of 2016, carcasses were thawed again for a secondary tissue dissection. At this time the combined cervix and vaginal wall were dissected free of the pelvic cavity, and these tissues were fixed in 4% paraformaldehyde in phosphate-buffered saline. Following overnight fixation these tissues were transferred to 70% EtOH, in preparation for shipping to the University of Kansas Medical Center.

### RNA isolation and quantitative RT-PCR analysis

Ovaries tissues were transferred directly from RNAlater to tubes containing 1 ml of Trizol (Sigma) or 1 ml of Trizol was added directly to ovarian tissue snap-frozen in liquid nitrogen. Tissues were briefly dissociated on ice (3 bursts, 62 W) in a PowerGen 700 homogenizer (Fisher Scientific). RNA was isolated from Trizol as per manufacturer protocol. To facilitate precipitation 1 μL of glycogen (20ug/ul; Invitrogen) was added to aqueous fraction and the resulting pellet was then resuspended in 15 μL of RNase free water (ThermoFisher 18064022). An aliquot (1 μL) of this resuspended RNA was used to determine RNA concentration and RNA integrity number (RIN) values using the Agilent RNA 6000 Pico kit and Agilent 2100 Bioanalyzer (Agilent Technologies). Total RNA (400 ng) from each sample was reverse transcribed using SuperScript II Reverse Transcriptase (Invitrogen/ThermoFisher) with random hexamer primers (IDT).

Quantitative PCR (qPCR) was performed using a 1:5 dilution of cDNA on an Applied Biosystems HT7900 sequence detector. Genes interrogated included 3β-hydroxysteroid dehydrogenase (*Hsd3b1*), P450 cholesterol side chain cleavage (*Cyp11a1*), 17α-hydroxylase (*Cyp17a1*), P450 aromatase (*Cyp19a1*), steroidogenic acute regulatory protein (*Star*), liver receptor homolog-1 (*Nr5a2*), estrogen receptor 1 (*Esr1*), estrogen receptor 2 (*Esr2*), scavenger receptor class B member 1 (*Scarb1*), luteinizing hormone receptor (*Lhcgr*), zona pellucida glycoprotein 3 (*Zp3*), and growth differentiation factor 9 (*Gdf9*). Primers were designed using Primer Express 3.0 (Applied Biosystems) and purchased from IDT (see Supplementary Table 1). qPCR was performed for *Hsd3b1*, *Cyp11a1, Cyp17a1, Cyp19a1, Nr5a2, Esr1, Esr2, Scarb1, Zp3, Lhcgr*, and *Gdf9* using PowerSYBR Green PCR Master Mix (Applied Biosystems). qPCR on *Star*, and 18 S rRNA levels were determined using the TaqMan Universal PCR Master Mix (Applied Biosystems) protocol and Applied Biosystems (Eukaryotic 18S rRNA Endogenous Control [VIC® ⁄ MGB Probe, Primer Limited]) probes and primers. Samples were run in triplicate, and the ΔΔCt method was used to calculate the relative expression between the samples after normalization with 18S levels. All SYBR Green reactions were evaluated for the presence of a single dissociation curve, to confirm the amplification of a single transcript and lack of primer dimers.

### Histological analysis

Upon receipt of tissues from the BSP, the vaginal walls samples were transferred to tissue embedding cassettes, and processed through a standard series of dehydration steps prior to being embedded in paraffin. Tissue blocks were then sectioned on a Leica microtome at a thickness of 6 μm and mounted on microscope slides (Superfrost Plus, Fisher Scientific). Tissue sections were then deparaffinized, rehydrated, and stained with hematoxylin and eosin prior to histological examination. Slides were randomized and sample (animal and treatment) labels were blinded prior to examination by three independent reviewers for estrous cycle staging (proestrus, estrus, metestrus, diestrus or not determinable). Non-determinable samples were the result of tissues not containing sufficient vaginal wall tissue to yield reliable results. Following independent reviews, those samples (~10%) without a consensus stage call were reevaluated by the reviewers and a consensus stage agreed upon.

### Radioimmunoassay

The steroid content (estrogen/progesterone) content of the ovary, as determined by RIA, was used as a surrogate for serum analyses for establishing the functional ovarian status of the RR1 females at the time of their sacrifice. Flash frozen ovaries were halved and homogenized using a sonicator in 200 μL of PBS. Homogenate was spun down at 10,000 × *g* for 5 min. Approximately 150 μL of supernatant was collected. 100 μL of undiluted supernatant was shipped to the Ligand Assay and Analysis Core (UVA School of Medicine) on dry ice and the rest were stored as 50 μL aliquots at −80 °C. Estradiol (E2) and progesterone (P4) were determined by commercial ELISAs (E2: Calbiotech, El Cajon, CA. Cat # ES180S-100; P4: IBL, Minneapolis, MN, Cat # IB79105). E2 assay characteristics were as follows: sensitivity = 3 pg/ml; intra-assay coefficient of variation (CV) = 7.5%; inter-assay CV = 10.1%. For P4, sensitivity = 0.15 ng/ml; intra-assay CV = 6.5%; inter-assay CV = 10.3%.

### Statistical analysis

One-way ANOVA was used to determine differences amongst treatment groups (flight status) or estrous cycle stage. Tukey’s mean separation tests were used to determine differences between mean once a significant *F*-test was observed. All statistical analyses and graphs for the RIA were performed using GraphPad Prism v6 (GraphPad, San Diego, CA).

### Reporting summary

Further information on experimental design is available in the Nature Research Reporting Summary linked to this paper.

## Supplementary information

Supplementary Figures and Tables

Reporting Summary Checklist

## Data Availability

Individual mouse estrous cycle stage results can be found in Supplementary Table 2.
