# Supplementary material for: Effects of spaceflight aboard the International Space Station on mouse estrous cycle and ovarian gene expression
Source: NPJ Microgravity. 2021 Mar 12;7:11. doi: 10.1038/s41526-021-00139-7 (PMC7954810; doi:10.1038/s41526-021-00139-7)
Supplement: Supplementary file 1 — Supplementary Figures and Tables [file 41526_2021_139_MOESM1_ESM.pdf]

Supplemental Figures and Tables

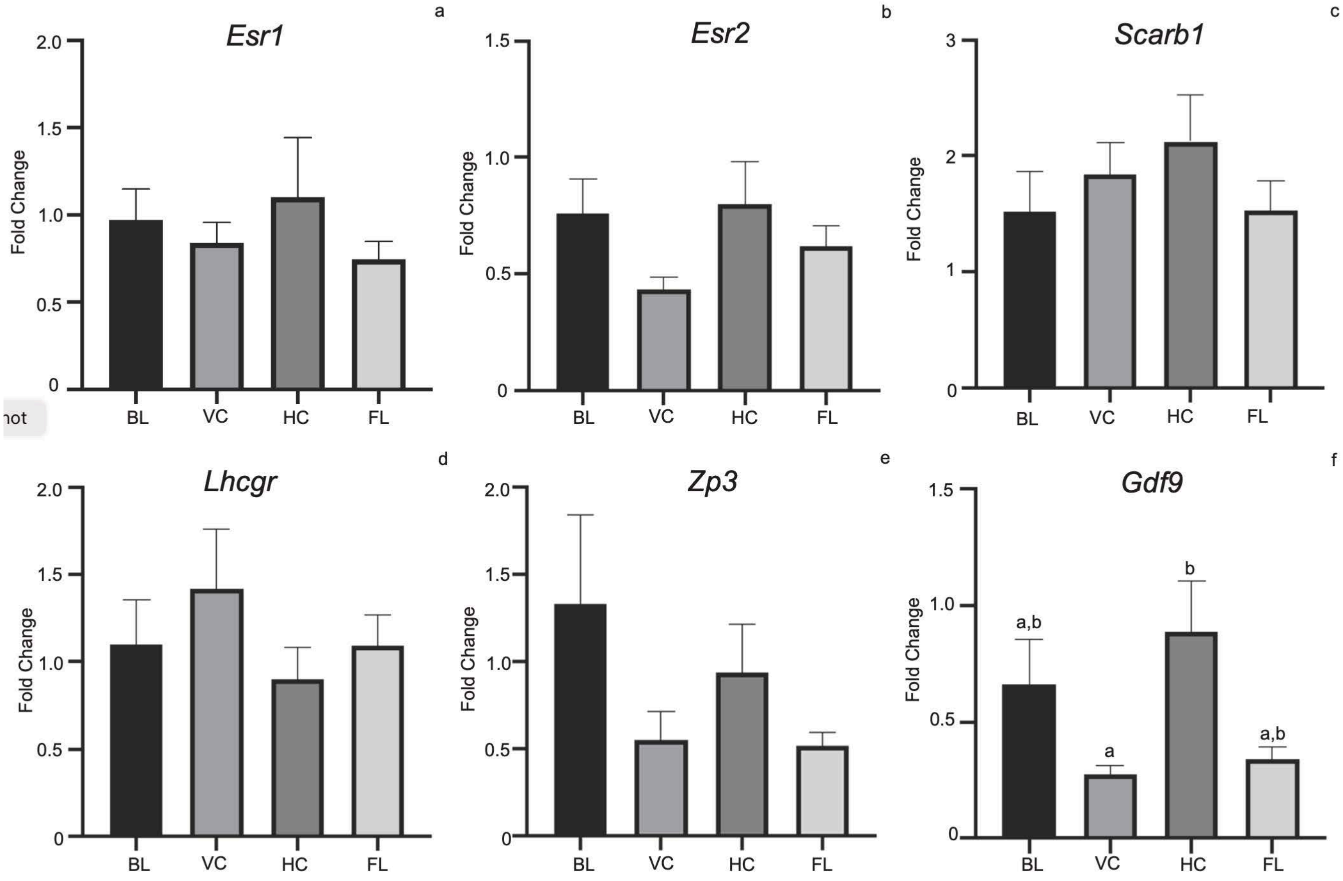

Supplemental Fig 1. Expression levels of important functional ovarian genes. Histograms depict expression levels for ESR1 (estrogen receptor alpha), ESR2 (estrogen receptor beta), SCARB1 (scavenger receptor class B member 1), Lhcgr (luteinizing hormone receptor), ZP3 (zona pellucida glycoprotein 3), GDF9 (growth differentiation factor 9). n=8-10/treatment group; 10BL, 10VC, 8HC, 10FL [a,b] Means ± SEM with different superscripts are significantly different (p≤0.05).

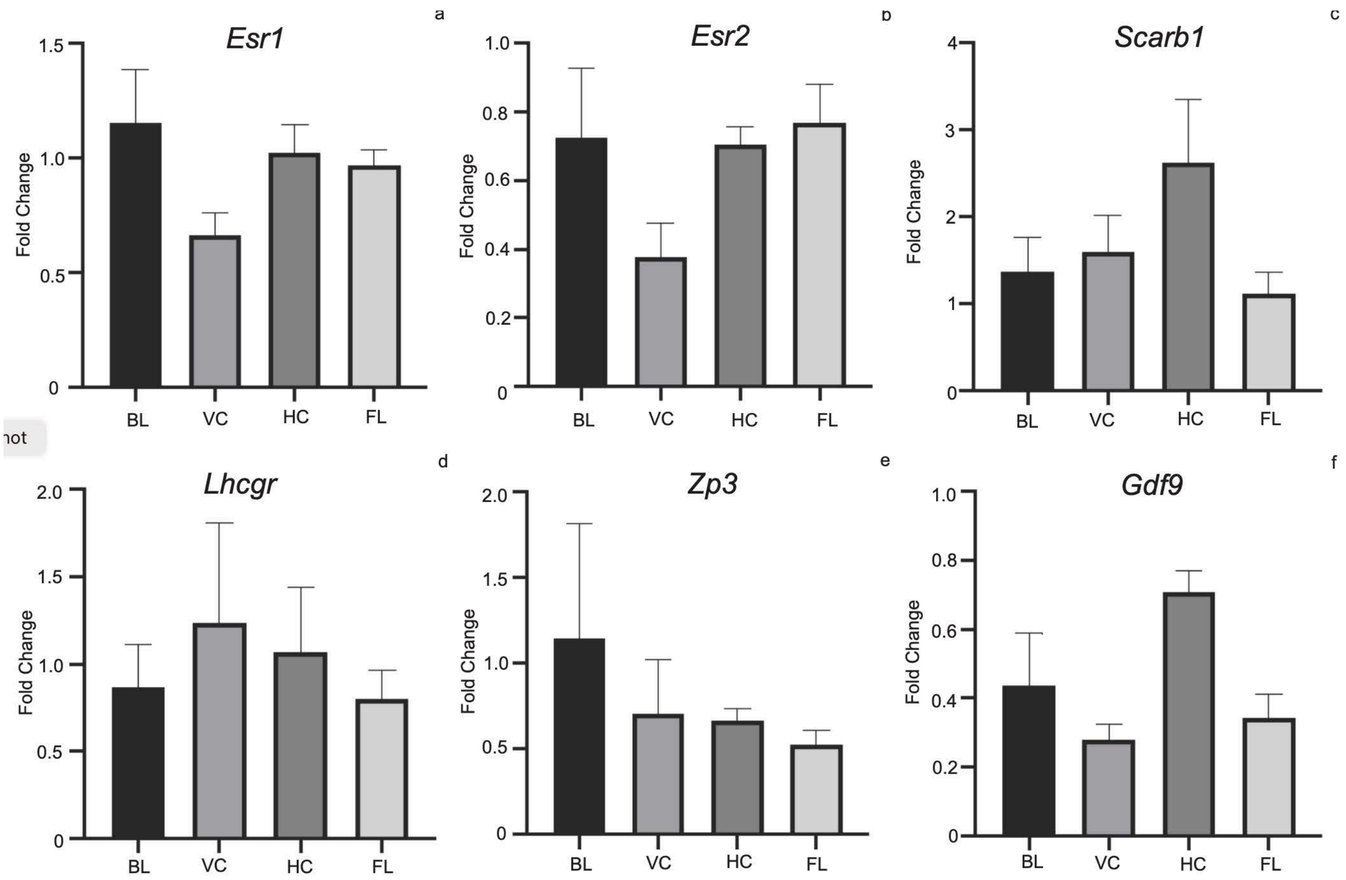

Supplemental Fig 2. Expression levels of important functional ovarian genes at estrus. Histograms show expression levels for ESR1 (estrogen receptor alpha), ESR2 (estrogen receptor beta), SCARB1 (scavenger receptor class B member 1), Lhcgr (luteinizing hormone receptor), ZP3 (zona pellucida glycoprotein 3), GDF9 (growth differentiation factor 9) during estrus. n=3-6/treatment group, 6BL, 5VC, 3HC, 6FL. No significant difference in expression levels were found.

Supplemental Table 1. Primers and TaqMan probes for Q-RT-PCR.

Gene	Primer - Forward / Reverse
3β-HSD	CCA GGC AGA CCA TCC TAG ATG
	TGG CAC ACT GGC TTG GAT AC
StAR	CCG GAG CAG AGT GGT GTC A
	GCC AGT GGA TGA AGC ACC AT
	Probe: CAG AGC TGA ACA CGG CCC CAC CT
P450 scc	CCA GTG TCC CCA TGC TCA AC
	GCA TGG TCC TTC CAG GTC TTA G
CYP17	TGG AGG CCA CTA TCC GAG AA
	TGT TAG CCT TGT GTG GGA TGA G
LHR-1	GGAACTGAAGGTTGATGACCAAA
	CGG TAA ATG TGA TCG AGA ATC AAG
Aromatase	TCA TAG CTC CTA TGG TTT GTC ATC A
	TCA CTG GTC CCC AAC ACA GA
ER 1	ATT CCG GAG TGT ACGT CT
	ACA CGG TGG ATG TGG TCC TT
ER 2	CCT GCC GAC TTC GCA AGT
	CCT TTC TCT CCT GGA TCC ACA CT

SCARB1	TGC CCA TGC CGA GAG TCT
	CAG AGG CGC ACC AAA CCT
ZP3	CAG GGT GCA GAT GAC GAA AGA
	CGA GGG TCG TGG AGT AGG AA
LHR	CTC GCT GGA GTT AAA AGA AAA CAT C
	TGG CCC CCT GGA AGG T
GDF9	CTA CAA TAC CGT CCG GCT CTT C
	GGT TAA ACA GCA GGT CCA CCA T

Supplemary Table 2 - RR1 Mice Identification and Experimental Group and Vaginal Wall Score/Stage of Estrous Cycle

Mouse ID	Exp Group	VW Score
M1	BL	ND
M2	BL	E
M3	BL	P
M4	BL	E
M5	BL	E
M6	BL	D
M7	BL	P
M8	BL	E
M9	BL	E
M10	BL	E
M11	VC	ND
M12	VC	E
M13	VC	E
M14	VC	P
M15	VC	E
M16	VC	P
M17	VC	E
M18	VC	E
M19	VC	P
M20	VC	ND
M21	FL	E
M22	FL	E
M23	FL	E
M24	FL	M
M25	FL	M
M26	FL	M
M27	FL	E
M28	FL	E
M29	FL	E
M30	FL	M
M31	HC	D
M32	HC	E
M33	HC	P
M34	HC	D
M35	HC	M
M36	HC	E
M37	HC	M
M38	HC	P
M39	HC	E
M40	HC	D
ND = no vaginal wall		
E = estrus		
P = proestrus		
M = metestrus		
D = diestrus		
